# Evaluation of the properties of a new super quick-setting (2 min) polyether impression material

**DOI:** 10.1007/s00784-023-04982-8

**Published:** 2023-04-05

**Authors:** Lamia Singer, Ludger Keilig, Michèle Fichte, Christoph Bourauel

**Affiliations:** 1 Oral Technology, Dental School, Medical Faculty, University Hospital of Bonn, Welschnonnenstr. 17, 53111, North Rhine-Westphalia, Bonn, Germany; 2 Department of Orthodontic, Dental School, Medical Faculty, University Hospital of Bonn, 53111, North Rhine-Westphalia, Bonn, Germany; 3 Department of Prosthodontics, Preclinical Education and Dental Materials Science, Dental School, Medical Faculty, University Hospital of Bonn, 53111, North Rhine-Westphalia, Bonn, Germany

**Keywords:** Elastomers, Dimensional accuracy, Tear strength, Super quick polyether, Polyvinylsiloxane

## Abstract

**Objectives:**

Although a new super-quick setting polyether impression material has been commercially recently introduced, its properties have not been yet reported. Thus, it was the aim of this study to assess the dimensional stability, tear strength, and elastic recovery of the new material and to compare it with another commonly used polyether and polyvinyl siloxane.

**Materials and methods:**

A new super-quick set polyether, a regular set polyether and a polyvinylsiloxane (PVS) impression material have been used in the study. Dimensional changes were measured using a modified mold as per ISO 4823:2000 after 1 h and 7 days. Tear strength was evaluated by subjecting specimens to tension until failure with a crosshead speed of 250 mm/min. Elastic recovery was measured by deforming specimens using a materials testing machine to a height of 16 mm (20% strain). The change in length (Δ*L*) was measured afterwards and elastic recovery was calculated in percentages.

**Results:**

Dimensional changes of the super quick and regular set polyether were comparable in both the vertical and horizontal dimensions after 24 h and 7 days. All the tested materials showed dimensional change values far below the maximum accepted ISO requirement (1.5%). The super quick setting polyether showed significantly improved tear strength (4.9 N/mm) in comparison to the regular set polyether (3.5 N/mm) and similar to PVS (5.2 N/mm). The elastic recovery of PVS (99.6%) was the highest among all the groups.

**Conclusions and clinical relevance:**

The newly available super-fast set polyether offers a great potential for a reduced chair side time and comfort for both, the patient and the dentist. Super quick polyether showed as well improved tear strength, which is considered one of the shortcomings of the regular set polyether. In addition, the new polyether was as accurate as the regular set polyether and with good elastic recovery.

## Introduction

An impression is a negative replica of the mouth’s soft and hard tissues which is typically taken for fabrication of indirect restorations [1]. Although selection of an appropriate impression material is very challenging, yet it is very important for an accurate and well-fitting prosthesis. Duplication of the intraoral structures is done using different materials ranging from hydrocolloids to elastomeric impression materials [2]. Elastomers are the most commonly used materials in everyday dental clinical practice for precise and accurate reproduction of oral cavity. Consequently, and until recently, we have been left with two good choices of elastomers, which are polyether (PE) and polyvinyl siloxane (PVS) [3].

Polyvinyl siloxane (PVS) or addition silicone was first introduced in the 1970s. PVS is a variation of condensation silicones in which they are both polydimethylsiloxane polymer, but with different terminal groups and thus different setting reactions [4]. PVS has inherently great dimensional stability, low polymerization shrinkage, high tear strength, and excellent elastic recovery [5]. On the other hand, PVS are naturally hydrophobic and therefore their uses are limited to cases where a dry environment could be obtained [6]. New modified hydrophilic polyvinyl siloxanes have been formulated afterwards, which can better flow, wet and record moist dental surfaces [7].

In 1965, polyether impression materials were introduced into the market in the form of a base and a catalyst. The base is made of polyether macro monomer with terminal ethylene imine rings, fillers, and plasticizers, while, the catalyst consists of dichlorobenzene sulfonate, thickening agents and colorants [8]. The polymer is formed during a cationic polymerization and opening of the imine rings, producing a cascade reaction that proceeds until polymerization stops. The backbone of the polymerized material is a copolymer of tetrahydrofuran and ethylene oxide with no reaction by-products resulting in a material with very good stability and accuracy [9].

PEs have an excellent hydrophilicity, flow, and were considered a vast improvement over hydrocolloids and condensation silicones in properties as tensile strength, and dimensional changes [10]. However, slow elastic recovery, stiffness and low tear strength are some of the drawbacks of the PE [11]. In 2000, efforts to overcome the shortcomings have led to the launch of an improved-taste, more flexible polyether impression materials (Penta Soft), that combines all the positive characteristics of polyether together with ease of handling [12].

Such improvement was achieved by decreasing the filler ratio to render a less rigid impression, and thus ease separation of impression from the mouth and the cast. Moreover, in 2005, a soft fast setting polyether impression material (6 min) was introduced through the addition of low-4 viscosity softeners to reduce the stiffness of the set PE [13, 14].

Nowadays, many impression systems and techniques are becoming more popular including hybrid materials with altered properties and intraoral digital impressions [15]. In numerous studies, it was reported that there is a comparable accuracy between digital scanners and conventional impressions in single crowns and short span bridges. However, they still show disadvantages compared to using conventional impression techniques with regard to longer spans or even full-arch rehabilitations [16, 17]. Conventional impression methods provide as well simpler way for dental cast production and allow easier laboratory adjustments [18]. Furthermore, the pervasive use of digital scanners is still limited due to high expenses and the need of special preparations that is sometimes challenging [15, 16].

However, and especially with respect to the partially quicker digital impression making, it is desirable that the impression materials cure within a shorter time span. This would reduce both the chair side time of the patient and the valuable time of the operator as well. The vast majority of the available polyether and silicone materials polymerize within 5–7 min, which is considered relatively long for single or small fixed prosthetic appliances [17]. To solve this issue, in 2020, a new PE material with a very fast working (0:45 s) and setting times (2:00 min) was very newly launched into the market. The objectives of the development of this material as claimed by the manufacturer is to combine the outstanding performance and accuracy of polyether with the fast-setting behavior of PVS [17].

Surprisingly, up until now, there is only one study in literature where the authors assessed the dimensional accuracy of the newly introduced material [17]. Moreover, there is no data available about the other physical and mechanical properties of the super quick set polyether although the manufacturer has claimed that they changed the composition to be able to decrease the setting time. Therefore, the aim of this in vitro study was to evaluate the dimensional accuracy, tear strength, and elastic recovery of the new super-fast setting impression material.

## Materials and methods

### Materials

Three commercially available elastomeric impression materials were used (Table 1).

**Table 1:** Materials used in the study

Impression materials	Super quick set polyether QSP	Regular set polyether RSP	Polyvinylsioxane PVS
Commercial name	Impregum Penta Super Quick Medium	Impregum Penta Medium	Express XT Medium
Manufacturer	3M ESPE^TM^ Deutschland GmbH Neuss, Germany	3M ESPE^TM^ Deutschland GmbH Seefeld, Germany	3M ESPE^TM^ Deutschland GmbH Neuss, Germany
Working time (min: s)	0:45	2:45	1:30
Setting time (min: s)	2:00	3:15	2:30
Mixing device	Pentamix 3	Pentamix 3	Pentamix 3
Batch Nr.	69385	31730	36894

### Methods

#### Dimensional changes

**Fig. 1:**
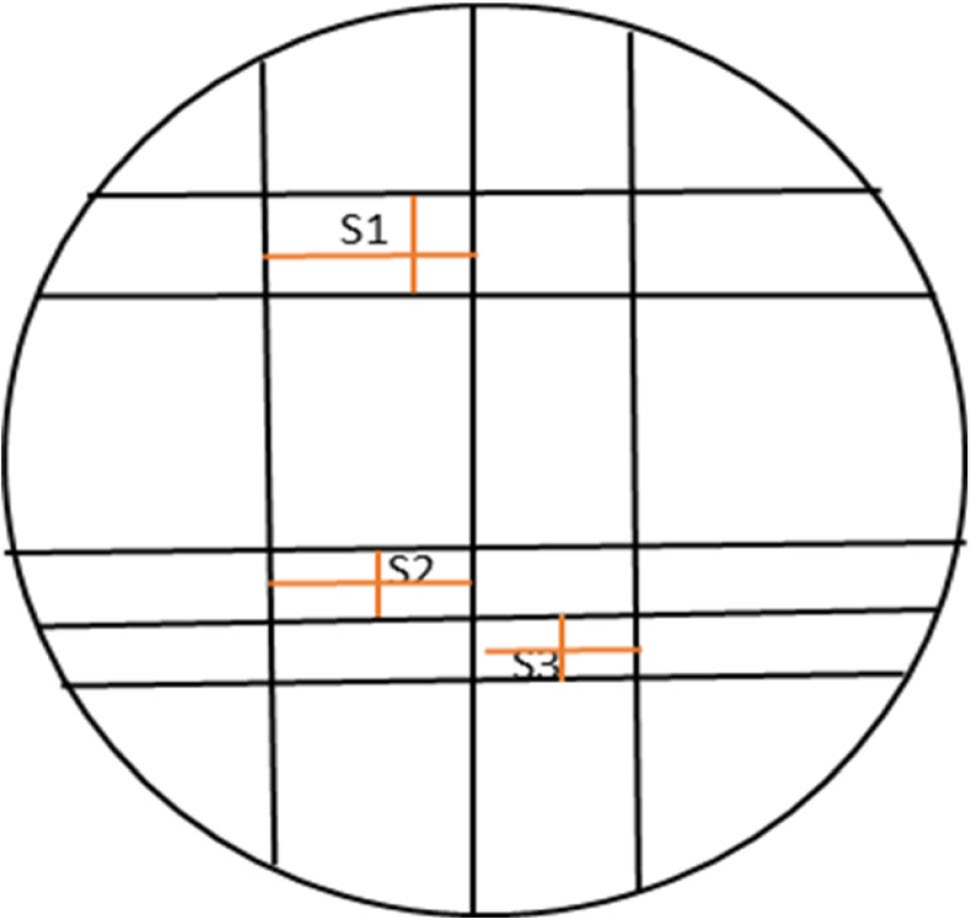
Schematic diagram of the dimensional change mold with vertical and horizontal measurements in the X and Y-axes (orange lines) at the top and bottom.

The test was conducted according to ADA [19] and ISO 4823:2000 protocols [20] but with slight modification of the specified metallic mold to allow measurements to be taken in the “X” and “Y” axes of different parts of the specimen. The modified mold consisted of three parts [21]:

- A ruled block with three vertical V-shaped lines (20-, 50-, and 75-μm width and 25 mm long) and five horizontal lines, 2 at the top and 3 at the bottom (resulting into three squares S1 (top), S2 and S3 (bottom; Fig. 1)).
- A metal ring to be fitted over the ruled block to provide a space and contain the material.
- A metal cover to obtain smooth flat material surface.

Before testing, the mold was washed and placed at 37 °C in an oven for 15 min in order to simulate the clinical situation. Both polyether materials and PVS were mixed according to the manufacturer instruction (*n*=10) using Pentamix 3 (Automatic mixer, 3M ESPE^TM^, Seefeld, Germany) equipped with the indicated mixing tips. The mold was filled with the homogenous mixture and covered with the metal plate. A 1-kg weight was placed over the metal plate to ensure tight sealing of the impression and to mimic the operator’s force exerted during impression making. The entire assembly was immersed in a water bath at 35 °C until the end of the setting time of each material as per the manufacturer’s recommendation.

As a perquisite inclusion criterion before testing, each sample was inspected at 6 X magnification using stereomicroscope (Leica Microsystems, Wetzlar, Germany) to confirm the continuity of the 75-μm line for every specimen. Every specimen was measured twice, 1 h after setting and after seven days of storage. The samples were stored in between the two measurements at 20±2 °C in a dry environment [22].

**Fig. 2:**
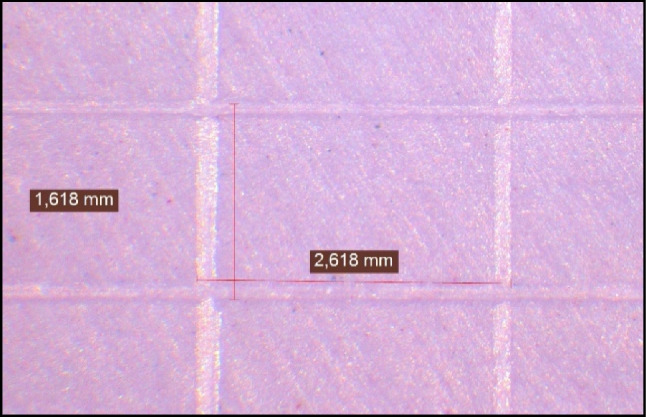
Measuring the vertical and horizontal dimensions of the upper part of regular set polyether sample under 12X for dimensional change assessment.

**Fig. 3:**
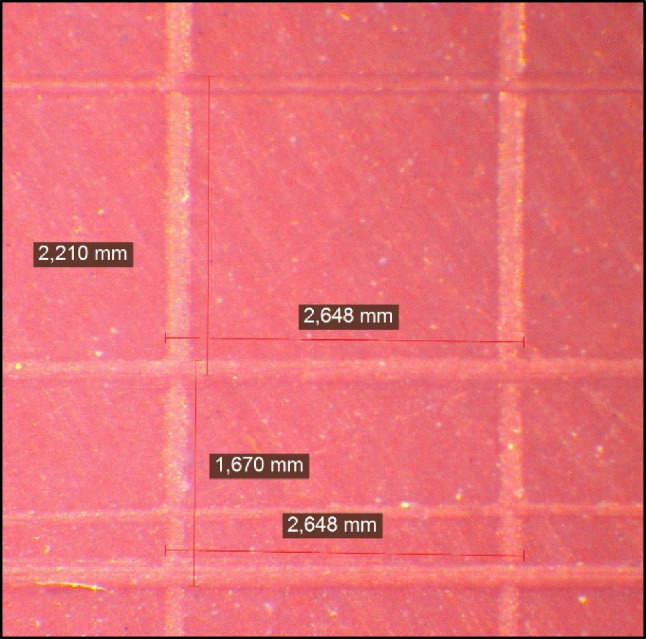
Measuring the vertical and horizontal dimensions of the lower part of super quick polyether sample under 12 X for dimensional change assessment.

The horizontal (H) and vertical dimensions (V) (in the X- and Y-axes) of the three squares S1, S2, and S3 at the top and bottom of each specimen were measured yielding six measurements in each sample (Figs. 2 and 3). Each dimension was measured three times and mean value of the measurements was calculated. Stereomicroscope (Carl Zeiss, Oberkochen, Germany) was used at a 12 X magnification. The same whole measurement technique was performed on the metallic mold without the impressions in order to determine and compare the dimensional changes.

The percentage of dimensional change was calculated for each specimen according to the formula presented by ISO 4823:2000 [20]:

$$\Delta L=\left(\ \frac{\textrm{L}1-\textrm{L}2\ }{\textrm{L}1}\right)\times 100$$

where *L*1 represents the distance measured of vertical or horizontal lines on the metallic mold (for S1+S2+S3) and *L*2 represents distance measured of vertical or horizontal lines (for S1+S2+S3) on the samples.

#### Tear strength

A plastic mold (96.4 mm length, 19.5 mm width and 13.7 mm thickness at the tearing point was 3D printed as recommended by American Society for Testing Materials (ASTM) specification for tear strength “Die C 12” [23]. The material to be evaluated was mixed (*n*=10) and dispensed inside the mold between two glass slabs. A silicone spray was used to facilitate separation of the specimens upon their polymerization. During setting, a 500 g weight was placed on the upper glass slab covering the mold to produce a smooth, flat specimen’s surface. Each specimen was carefully inspected and excess material of the prepared specimens was meticulously trimmed. Three areas of each specimen narrow portion were measured three times using a digital calliper (Mitutoyo, Tokyo, Japan) to accurately confirm width and thickness. Dimensions were then averaged to obtain a final measurement. Specimens that were not in accordance with the dimensions specified within the DIN 53504 were discarded [24].

**Fig. 4:**
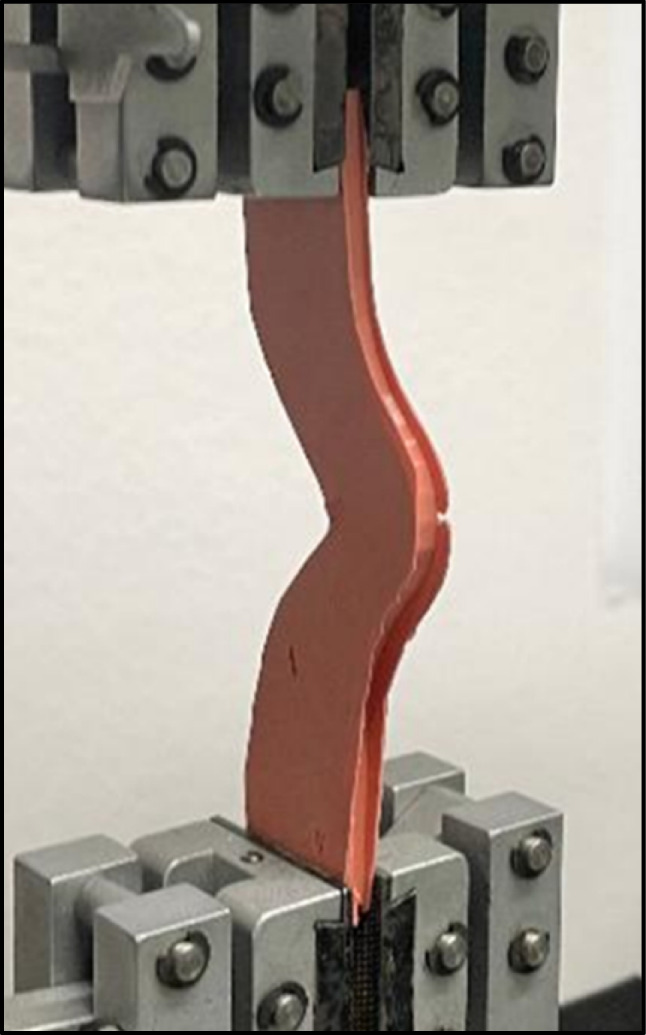
Tear strength specimen

According to the storage time (1 h or 7 days after setting), prepared samples were secured into a Zwick universal testing machine (Zwick Zmart Pro, Zwick Roell GmbH & Co. KG, Ulm, Germany). Each specimen was gripped from both edges by a mechanical clamp and the jig was adjusted so that the specimen was neither in compression nor tension (Fig. 4). Specimens were loaded in tension until rupture with a crosshead speed of 250 mm/min [25, 26]. The load at rupture was used to determine the tear strength according to the following equation: *T*=*F*\*d,* where *T*: tear strength in N/mm, *F*: tearing force, *d*: thickness of the specimens.

#### Elastic recovery

A mold formed of a fixation ring (20.5 mm inside diameter, 19 mm height) and a split plastic mold (12.5 mm inside diameter, 20.5 mm outside and 20 mm height) was used to prepare elastic recovery specimens as per ISO 4823 [20]. Each material was mixed as per manufacturer’s instructions, placed inside the mold and a glass plate was pressed on the top to remove the excess and to form a flat smooth surface. The assembly was immersed in a water bath (36±1 °C) until the end of the known initial setting time of each material.

**Fig. 5:**
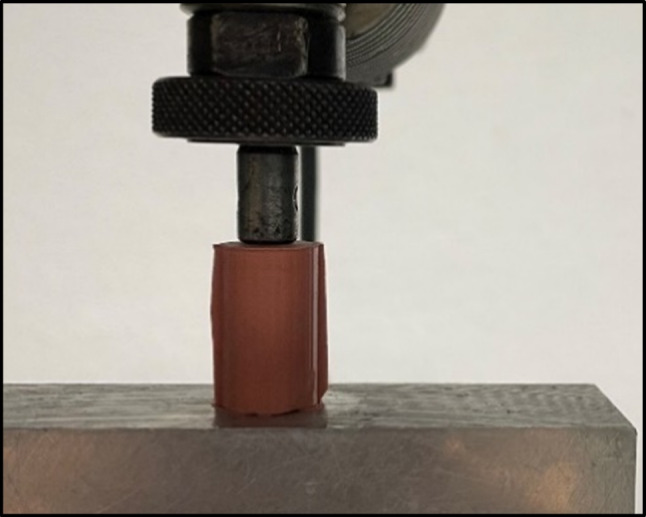
Deformation of PVS specimen using Zwick testing machine

Specimens were examined 6 min after removal from the water bath. Each specimen was checked and measured using a digital micrometer, the initial reading was recorded as reading L in mm. Five seconds later, the specimen was deformed using a Zwick testing machine to a height of 16 mm (20% strain) within 4 s and the deformation was maintained for 5 s and then released (Fig. 5). Thirty seconds after the release, the specimen was measured again and to record the change in length Δ*L* was measured and strain in compression was calculated as follows:

$$\textrm{Elastic}\ \textrm{recovery}=\left(\frac{\Delta L}{L}-1\right)\times 100$$

where *L* is the height of the sample before compression and Δ*L* is the change in length.

#### Statistical analysis

Numerical data were presented as mean and standard deviation (SD) values. Only dimensional changes data was presented as medians as they showed non-parametric distribution, so Kruskal-Wallis test was used for comparison between the three groups. All other quantitative variables showed parametric distribution; thus, one-way analysis of variance (ANOVA) was used for comparison between the groups. Tukey’s post hoc test was used for pair-wise comparison when ANOVA test was significant. The significance level was set at *p*≤ 0.05. Statistical analysis was performed using Minitab 17.1.0 for Microsoft Windows.

## Results

### Dimensional accuracy

**Fig. 6:**
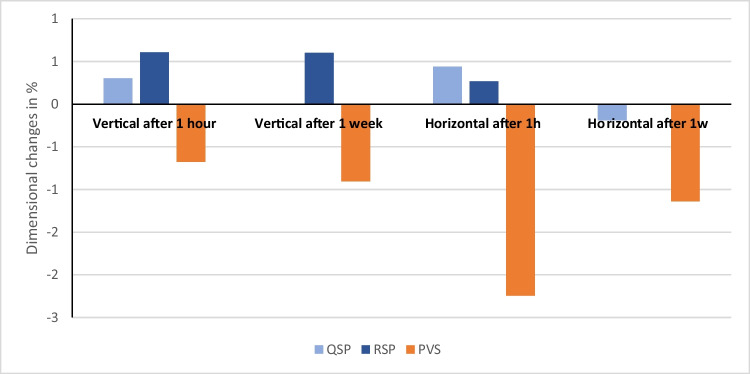
Bar chart showing dimensional change values in percentages for the three tested materials after 1 h and 1 week in X and y axes

**Fig. 7:**
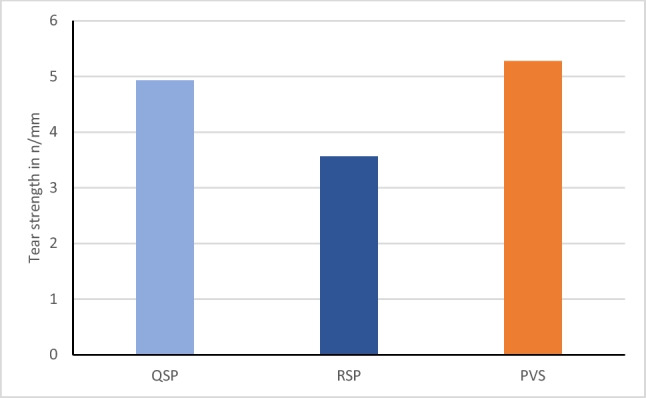
Bar chart representing the mean tear strength values of the three tested impression materials.

Data showed non-parametric distribution, and thus, numerical values of the median and standard deviation of the dimensional changes of the tested materials are listed in Tables 2, 3, 4, and 5 and represented in Figs. 6 and 7. Based on the findings, there was a statistically significant difference in dimensional changes between the three tested materials after 24 h and 7 days.

**Table 2:** Median, standard deviation (SD) values, and results for vertical dimensional changes in percentage for the tested materials after 24 h

Groups	*N*	Median %	Confidence interval		*p*-value	Across groups
			Lower	Upper		
QSP	30	0.30	0.00	1.24	<0.01	A
RSP	30	0.61	0.30	0.98		A
PVS	30	−0.68	−1.11	−0.18		B

**Table 3:** Median, standard deviation (SD) values, and results for horizontal dimensional changes in percentage for the three tested materials after 24 h

Groups	*N*	Median %	Confidence interval		*p*-value	Across groups
			Lower	Upper		
QSP	30	0.44	0.11	0.62	<0.01	A
RSP	30	0.27	0.11	0.61		A
PVS	30	−2.24	−2.27	−1.83		B

**Table 4:** Median, standard deviation (SD) values, and results for vertical dimensional changes in percentage for the three tested materials after 1 week

Groups	*N*	Median %	Confidence interval		*p*-value	Across groups
			Lower	Upper		
QSP	30	0.00	0.00	1.21	<0.05	AB
RSP	30	0.60	0.00	1.23		A
PVS	30	−0.90	−1.81	0.04		B

**Table 5:** Median, standard deviation (SD) values, and results for horizontal dimensional changes in percentage for the three tested materials after 1 week

Groups	*N*	Median %	Confidence interval		*p*-value	Across groups
			Lower	Upper		
QSP	30	−0.19	−0.38	0.00	<0.01	A
RSP	30	0.00	−0.38	0.00		A
PVS	30	−1.14	−1.16	−0.76		B

### Dimensional changes after 24 h

The sum of the vertical dimension measurements (S1+S2+S3; Table 2) for the regular set polyether showed the highest median dimensional changes (*M*=0.61%). However, the new super quick set polyether (QSP) showed lower median dimensional changes (*M*=0.30%) compared to regular set PE (RSP), whereas PVS showed statistically negative median values (*M*=−0.68%). Statistically, vertical dimension measurements on QSP specimens differed significantly from both other materials, while no statistically significant difference was found between PE and PVS. For the horizontal dimensions (Table 3), the new super quick set PE (*M*=0.44%) and the regular PE (*M*=0.27%) did not differ significantly from each other but significantly different from PVS that showed the highest dimensional changes (*M*=−2.24%).

### Dimensional changes after 1 week

Statistical analysis of the sum of the vertical dimension measurements (S1+S2+S3) after 1 week (Table 4) showed that the regular set polyether (*M*=0.60%) and the new super quick set polyether (*M*=0.00%) did not differ significantly. PVS (*M*=−0.81%) showed negative results that was not significantly different from quick PE but significantly different from regular PE. For the horizontal dimensions (Table 5), the new super quick set PE (*M*=−0.19%) and the regular PE (*M*=0.00%) did not differ statistically from each other but were both significantly different from PVS (*M*=−1.16%).

### Tear strength

The variables showed parametric distribution, and thus tear strength values were analyzed by one way ANOVA followed by Tukey’s post hoc for pairwise comparison between the different groups. The means and standard deviations of the tear strength in N/mm are illustrated in Fig. 7. Results revealed that there was statistically significant difference between tear strength of the three materials (*p*-value =0.000). Regular set PE showed mean tear strength of 3.55 (SD=0.26) N/mm, whereas each of super quick set PE and PVS recorded significantly higher mean tear strength values of 4.92 (SD=0.93) N/mm and 5.27 (SD=0. 67) N/mm respectively.

### Elastic recovery

**Fig. 8:**
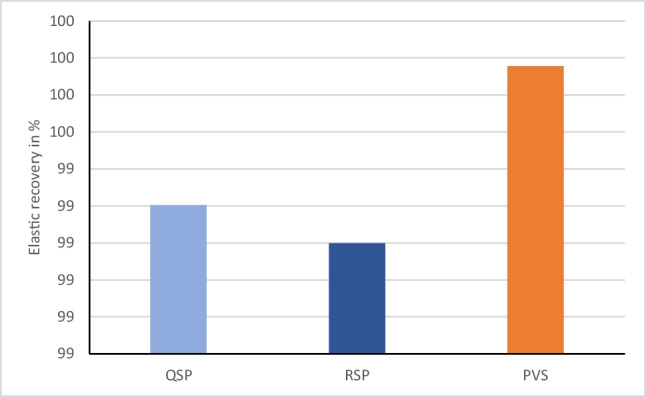
Bar chart representing the mean elastic recovery in percentages for the three tested impression materials

Statistical analysis of the means and standard deviations of recovery from deformation (in percentage) for the three tested impression materials are represented in Fig. 8. One-way ANOVA indicated that there was statistically significant difference in the elastic recovery of the three materials (*p*-value=0.011). PVS recorded a mean elastic recovery of 99.6% (SD=0.16) which was significantly higher from both the super quick set PE (99.3%, SD= 0.33) and the regular set one (99.1%, SD=0.47). All of the materials tested met the ISO4823 requirement of having greater than 96.5% recovery.

## Discussion

### Dimensional changes

The dimensional changes of impression materials may affect the retention and adaptation of final indirect dental restoration. In addition, dimensional stability over a long period is a very important characteristic, which permits the production of precise cast models at any time [27]. Several factors influence the dimensional behavior of impression material such as humidity, working time, and flow and thickness of the material inside the tray [28].

There are common methods applied to evaluate the dimensional stability of impression materials including; direct measurement of the impressions, comparison between the master and plaster models, and assessment of the fit of restorations over the plaster cast. In the present study, dimensional accuracy and stability were assessed directly on the impression after 1 h and 7 days storage to simulate a delay between impression taking and digitizing or casting. Stereomicroscope was used for horizontal and vertical measurements.

Most studies follow protocols described by the American Dental Association (ADA) and ISO 4823, which replicates a clinical scenario [19]. These guidelines recommend using a cylindrical metal block allowing measurements over two horizontal coordinates in an area of less than 5mm in length [29]. In the current study, measurements were taken over three horizontal and three vertical coordinates.

Results showed that there was statistically significant difference between the PVS group (*V*= −0.68%, *H*=−2.24%) and both the regular set polyether (*V*=0.61%, *H*=0.27%) and the super quick set (*V*=0.30%, *H*=0.44%) after 24 h in the vertical and horizontal dimensions. All changes were far below the limits reported by ISO International standard 4823 specification of less than the 1.5% after a minimum of 24 h except for the median horizontal values of PVS (*M*=−2.24%) [20, 30]. In the present study, simulation of the mouth temperature was performed by preheating the metal mold; thus, positive results could be explained based on the linear expansion thermal coefficient, in which impression materials contract upon removal from the mouth due to temperature difference with the extra oral environment [28].

Data showed that measurements between impression materials were different as well with time (after 1 week). There were fluctuations in measurements mostly expansion within all the tested impression materials in the vertical (except PVS) and horizontal dimensions. Expansion (negative results) is most probably related to hygroscopic expansion or residual stress relaxation [31]. The expansion compensated for some of the shrinkage happened and therefore, improved the accuracy in the horizontal and vertical dimensions. These data contradict Piwowarczyk et al. in 2002 [32] who concluded that no significant dimensional changes occurred between the different elastomeric impression materials over different time intervals.

Polyether polymerizes via a reaction between the aziridine rings located at the end of the branch of its own molecules, and cross-linking is initiated by an aromatic ester sulfonate. In this reaction, no sub-products are released, which favors the dimensional accuracy and stability of the impression [9]. However, unlike other materials, the high hydrophilic characteristic of polyether can lead to the absorption of water from the atmosphere and from the storage medium [33]. This material shows a greater stability over time when compared to polysulfide and condensation silicone, despite controversy regarding the time of pouring whether immediate or periods of up to 24 h [34–36] and 1 week [37].

Addition silicones also release negligible number of by-products thus the material undergo little dimensional changes and is considered stable [38]. Results of the present study are in accordance with scientists who proved that the duration of storage of PVS affects the dimensional changes [39, 40]. On the other hand, the results contradict studies, which stated that addition silicone dimensional stability is not time dependent and casting can be done several times without the loss of materials properties [41, 42].

### Tear strength

Dental impressions should resist tensile tearing stresses upon removal from the mouth and upon cast separation from the set impression [43]. The clinical tear performance of a material relies on complex interactions between the fillers and the polymer, thickness of the material, presence of internal voids, surface defects, and the removal rate. Because of the difficulties of integrating and measuring all of these properties, laboratory tests assessing the propagation energy of a tear have been employed for elastic dental materials [44].

Moreover, setting time of the material is strongly correlated to its tear strength. Shorter setting times are more convenient for clinicians and patients, but if the setting time is too short and the material has not completely polymerized before removal, the impression material will tear [23]. The ANSI/ADA standard states that tear strength should be measured 1 h following the manufacturer’s setting time [19] although impressions are subjected clinically to tearing forces immediately after setting. Therefore, in the present study, tear strength was measured immediately after setting to mimic the clinical situation.

Results of the current study showed that the tear strength of the new super-quick setting polyether (4.9 N/mm) and polyvinylsiloxane (5.2 N/mm) were comparable to each other but they were significantly higher than the regular setting polyether (3.5 N/mm). This was in agreement with results obtained by Lawson NC et al. [23] and Dino et al. [45], who stated that addition silicone materials provided higher tear strength than polyether materials. Nevertheless, the results contradict Lu et al. who confirmed that PE impression materials had higher tear energy in compression compared to new hydrophilic addition silicone materials [46].

The manufacturer of the new super-fast setting polyether claims that they have modified the composition and initiator system to speed up the setting time. Thus, this could be a reason for the significant change between the regular set polyether and the new one and the non-significant difference in behavior with the PVS. Moreover, the higher tear strength of the new super-quick material could be due to some reduction of the filler amount compared to the regular set one, which is the same approach that has been taken by the manufacturer before to produce a softer material [47].

Additionally, the hydrophilicity or hydrophobicity levels of impression materials usually affect the tear strength property. The incorporation of oral fluids during polymerization might results in defects that act as stress initiators, reducing the tear strength of the polymerized material [48]. On the other hand, polyvinylsiloxanes deform at much slower rates and tear at points of less permanent deformation than do the other elastomeric impression materials [48], and they are less rigid than polyether when set [30, 33].

Many studies [33, 49, 50] have been carried out on tear strength though; there is no single test method has been standardized yet. Consequently, comparing different impression materials using the existing literature information still considered quite difficult; besides, there is still no sufficient data about the new fast setting material.

### Elastic recovery

Dental impression materials should preserve its elastic behavior when subjected to stresses in tissue undercuts and deep grooves [13]. The distortion of an impression material past its elastic range may cause permanent deformation and renders it inaccurate. Impression materials are polymers with highly flexible coiled chains that uncoil upon loading and exhibit nearly complete elastic recovery when the load is removed [35]. Permanent deformation is related to factors such as the degree of cross-linking of the polymer strands, temperature, and the rate of applied stress [40, 44]. Lu et al. [46] found that flexibility or stiffness of the material was inversely correlated to elastic recovery; therefore, the higher the elastic recovery, the lower the stiffness.

Elastic recovery in the present study was tested by compression set rather than tension. Blomberg et al. [51] reported a strong correlation between elastic recovery from tensile and compressive strain and therefore reported that only one method is necessary. All of the materials in this study met the requirement of ISO 4823 of having elastic recovery higher 96.5% [20]. The results showed that the mean elastic recovery of polyvinylsiloxane was (99.6%), which was significantly higher than the super-quick setting polyether (99.3%) and the regular setting polyether (99.1%). The differences between the two types of polyether were not statistically significant.

These results are in accordance with a study, which reported that polyvinylsiloxanes have sufficient elastic recovery to allow an impression to be poured only 6 min after removal from the mouth [51]. For PVS materials, the elastic recovery is dependent on components, such as base silica, copolymer filler, and chain extenders [52]. Moreover, polyvinylsiloxanes have the least viscoelastic qualities thus requiring the least time for recovery from viscoelastic deformation [48]. Results matches those of Donovan et al. [27] which concluded that PVS exhibit the best elastic recovery, followed by polyether and polysulfide.

## Conclusions

The super-quick setting polyether performed comparable to PVS with regard to tear strength and at the same time was as accurate as the regular set PE. All of the materials in this study met the requirement of ISO 4823 standard, which requires greater than 96.5% recovery. Thus, the 2-min setting material offers a promising prospective to save the valuable time of the dentist and provide more comfort to the patient without compromising the quality of the final restoration.

## Data Availability

The datasets used and/or analyzed during the current study are available from the corresponding author on reasonable request.
